# Micro/Nanoarchitectonics of 3D Printed Scaffolds with Excellent Biocompatibility Prepared Using Femtosecond Laser Two-Photon Polymerization for Tissue Engineering Applications

**DOI:** 10.3390/nano12030391

**Published:** 2022-01-25

**Authors:** Yanping Yuan, Lei Chen, Ziyuan Shi, Jimin Chen

**Affiliations:** 1 Faculty of Materials and Manufacturing, Institute of Laser Engineering, Beijing University of Technology, Beijing 100124, China; chenlei@emails.bjut.edu.cn (L.C.); shiziyuan@emails.biut.edu.cn (Z.S.); jimin@bjut.edu.cn (J.C.); 2 Key Laboratory of Trans-Scale Laser Manufacturing Technology, Beijing University of Technology, Ministry of Education, Beijing 100124, China; 3 Beijing Engineering Research Center of 3D Printing for Digital Medical Health, Beijing University of Technology, Beijing 100124, China

**Keywords:** micro/nano 3D printed scaffold, femtosecond laser, two-photon polymerization

## Abstract

The fabrication of high-precision scaffolds with excellent biocompatibility for tissue engineering has become a research hotspot. Two-photon polymerization (TPP) can break the optical diffraction limit and is used to fabricate high-resolution three-dimensional (3D) microstructures. In this study, the biological properties, and machinability of photosensitive gelatin methacrylate (GelMA) hydrogel solutions are investigated, and the biocompatibility of 3D scaffolds using a photosensitive GelMA hydrogel solution fabricated by TPP is also evaluated. The biological properties of photosensitive GelMA hydrogel solutions are evaluated by analyzing their cytotoxicity, swelling ratio, and degradation ratio. The experimental results indicate that: (1) photosensitive GelMA hydrogel solutions with remarkable biological properties and processability are suitable for cell attachment. (2) a micro/nano 3D printed scaffold with good biocompatibility is fabricated using a laser scanning speed of 150 μm/s, laser power of 7.8 mW, layer distance of 150 nm and a photosensitive GelMA hydrogel solution with a concentration of 12% (*w*/*v*). Micro/nano additive manufacturing will have broad application prospects in the tissue engineering field.

## 1. Introduction

Excellent scaffolds for tissue engineering should be three-dimensional (3D) porous structures that maintain the shape and characteristics of lesion tissues [1,2,3]. In addition, the materials used to fabricate scaffolds for tissue engineering should present excellent biocompatibility, high water absorption capacity, and remarkable biodegradability [4,5]. Scaffolds used for soft tissue engineering, such as cartilage, tendon, skin, blood vessel, kidney, and nerve tissue, are typically fabricated from photosensitive solutions or bioinks prepared using synthetic hydrogels. Synthetic hydrogels are fabricated using hydrophilic groups and high-molecular polymers [6,7]. Owing to the cytotoxicity of polymers, synthetic hydrogels present poor biocompatibility, low water absorption capacity, and poor biodegradability, which severely limit their application for tissue engineering scaffolds. Hence, natural hydrophilic materials modified using organic groups, such as naturally derived hydrogels, have attracted increasing attention. Naturally derived hydrogels [8,9] such as collagen, sodium alginate, chitosan, gelatin methacrylate (GelMA), and composite hydrogels, which can easily undergo cross-linking reactions, have been widely used for tissue engineering.

In addition, the structure of the scaffolds utilized for tissue engineering, such as porosity, is also of great concern. Several researchers have focused on the pore-forming ability of scaffolds and internal pore structure of scaffolds [10,11,12]. However, scaffolds present relatively simple shapes, such as cylinder, cube, and thin film shapes. Hence, traditional methods are ineffective for fabricating complex external structures. Additive manufacturing (AM) technology is based on the layer-by-layer fabrication principle, which renders it advantageous for fabricating complex 3D porous scaffolds. Moreover, AM facilitates the fabrication of customized scaffolds. Inkjet, extrusion, and photopolymerization are the types of AM technologies most commonly used to fabricate tissue engineering scaffolds using naturally derived hydrogels. Inkjet and extrusion do not require photoinitiators, which reduces the cytotoxicity of the fabricated materials. Researchers have successfully fabricated 3D scaffolds for cartilage [13], skin [14], muscle [15], vessel [16], heart [17,18], nerve [19], stem cell [20], periodontal [21] and corneal [22] tissue engineering. However, the precision of 3D scaffolds fabricated using inkjet and extrusion technologies is poor because processing resolution is low. Based on the cross-linking photopolymerization reaction, stereolithography (SLA), digital light processing (DLP), digital micromirror device (DMD), and two-photon polymerization (TPP) technologies require initiation using external light sources. Hence, photosensitive hydrogel solutions should be configured using photoinitiators. SLA, DLP, and DMD technologies have been successfully used to fabricate 3D porous scaffolds for liver [23], nerve [24], vascular [25,26] and stem cell [27] tissue engineering. Owing to their relatively high processing resolution, the aforementioned 3D porous scaffolds are relatively high precision. TPP technology [28] is not diffraction limited and can use for sub-wavelength fabrication. Structures fabricated by TPP technology have dimensions below 100 nm. Most commercial photoinitiators are sensitive to light sources with wavelengths of 365–450 nm. Therefore, they are usually used in SLA, DLP, and DMD technologies. However, commercial photoinitiators have small two-photon absorption cross sections. The TPP effect is typically induced in naturally derived hydrogels using symmetric structure [29] photoinitiators with a large two-photon absorption cross section.

In this study, we used a femtosecond laser with a wavelength of 780 nm to induce the TPP effect. Most commercial photoinitiators require UV light sources, and their absorption cross-section in the near-infrared region is very small. Hence, photosensitive GelMA hydrogel solutions were configured using a combination of GelMA hydrogel, photoinitiator, and photosensitizer, to improve absorption efficiency in the near-infrared region (~780 nm). The biological properties of the photosensitive GelMA hydrogel solutions, namely cytotoxicity, swelling ratio, and degradation ratio, were evaluated. Moreover, the processability of photosensitive GelMA hydrogel solutions was evaluated by using them to fabricate 3D structures. The experimental results indicated that the photosensitive GelMA hydrogel solutions presented good biocompatibility, swelling ratio, degradation ratio, and processability. A micro/nano 3D printed scaffold with good biocompatibility was fabricated using a laser scanning speed of 150 μm/s, laser power of 7.8 mW, layer distance of 150 nm, and a photosensitive GelMA hydrogel solution with a concentration of 12% (*w*/*v*). We anticipate that micro/nano 3D AM will have broad application prospects in the tissue engineering field.

## 2. Materials and Methods

### 2.1. Configuration of Photosensitive GelMA Hydrogel Solutions

In this study, a 780 nm femtosecond laser was used to induce the TPP effect. However, most commercial photoinitiators require UV light sources, and their absorption cross-sections in the near-infrared region are very small. Hence, Rose Bengal was selected as the photosensitizer to improve the absorption efficiency of the VA-086 photoinitiator in the near-infrared region (~780 nm). Dried GelMA hydrogel (Hangzhou, China) was added to 1× phosphate buffered saline (PBS; Solarbio, Beijing, China) solution. GelMA hydrogel solutions with concentrations of 5%, 8%, 10%, 12%, 15%, and 20% (*w*/*v*) were configured. Thereafter, a 1% (*w*/*v*) VA-086 (Wako Pure Chemicals, Osaka, Japan) photoinitiator and a 1% (*w*/*v*) Rose Bengal (Sigma-Aldrich Chemical Co., St. Louis, MO, USA) photosensitizer were added to the GelMA hydrogel solution. Subsequently the mixtures were heated in a 37 °C water bath for 10 min, followed by shaking in an ultrasonic shaker for 10 min to ensure that the GelMA hydrogel fully dissolved in the PBS solution. Subsequently, a 0.22 μm needle filter (SLGV033RB, Millipore, Atlanta, GA, USA) was used to filter the solutions. Next, the configured photosensitive GelMA hydrogel solutions comprising GelMA hydrogel, VA-086 photoinitiator, and Rose Bengal photosensitizer were used to fabricate 3D scaffolds.

### 2.2. Biological Properties of GelMA Hydrogel Solutions

Cytotoxicity: To comprehensively assess the cytotoxicity of the prepared solutions, samples with a diameter of 6 mm and a thickness of 2 mm were polymerized using the prepared photosensitive GelMA hydrogel solutions. The samples were soaked in PBS to obtain soaking solution. The cytotoxicity of the soaking, photosensitive GelMA hydrogel and photoinitiator solutions were tested [30,31].

The Cell Counting Kit−8 (CCK−8) method was used to evaluate the cytotoxicity of the soaking, photosensitive GelMA hydrogel and photoinitiator solutions. HPF-1 human lung fibroblasts were selected and seeded in a 96-well microtiter plate (3599, Corning Costar, Alexandria, VA, USA) at a density of 5000 cells per well. The seeded cells were incubated for 24 h at 37 °C under a 5% CO_2_ atmosphere. Thereafter, the Dulbecco’s Modified Eagle Medium was removed from the wells, and the soaking, photosensitive GelMA hydrogel and photoinitiator solutions were added to the wells. Subsequently, the 96-well microtiter plate was placed in a cell incubator (Forma Steri-Cycle, Thermo Scientific, Waltham, MA, USA) for 3 h. The CCK−8 reagent (Dojindo, Kumamoto, Japan) was added to the wells, and then the 96-well microtiter plate was placed in a cell incubator for 1 h. The absorbance of the wells was measured using a multilabel plate reader (Victor 3 V 1420, Perkin Elmer, Boston, MA, USA), and cell activity was calculated using the absorbance to assess the cytotoxicity of the GelMA hydrogel solutions.

Swelling ratio: As the 3D structures fabricated using the TPP method are too small to be weighed using conventional weighing methods, macroscopic samples were used to measure the swelling ratio [4] of the polymerized samples. Samples with a diameter of 6 mm and thickness of 2 mm were polymerized using the prepared configured photosensitive GelMA hydrogel solutions and a light source with a center wavelength of 405 nm. The polymerized samples were freeze-dried, and weighed using an electronic analytical balance (Pioneer, Ohaus, Parsippany, NJ, USA). After the dry weight ($W_{\mathrm{dry}}$) of each sample was recorded, the samples were soaked in a PBS solution at 18−22 °C for 1, 2, 3, 6, 9, 12, and 24 h. Thereafter the soaked samples were taken out from the PBS solution, the surface water was removed, the samples were weighed again, and their weight ($W_{\mathrm{swollen}}$) was recorded. Lastly, the swelling ratios of the samples were calculated as follows:

(1) $$\mathrm{Swelling}\;\mathrm{ratio}\;\left(\%\right)=\frac{W_{\mathrm{swollen}}-W_{\mathrm{dry}}}{W_{\mathrm{dry}}}\times 100,$$

Degradation ratio: In vitro degradation rates were evaluated by measuring percent mass loss. Before degradation studies all polymerized samples were lyophilized and weighed to determine their initial weight ($W_{t0}$). A protease solution with a concentration of 4.2 units/mL was used to degrade the samples. After soaking them in the protease solution at 37 °C for 1, 2, 3, 6, 9, 12, 24, 36, and 48 h, the samples were removed from the protease solution, the surface water was removed, the samples were weighed, and their weight $(W_{\mathrm{tDeg}}$) was recorded. The degradation ratios of the samples were calculated as follows:

(2) $$\mathrm{Mass}\;\mathrm{loss}\;\left(\%\right)=\frac{W_{t0}-W_{\mathrm{tDeg}}}{W_{t0}}\times 100,$$

where $W_{t0}$ is the original dry mass of the sample, $W_{\mathrm{tDeg}}$ is the residual dry mass of the sample after a degradation period.

### 2.3. TPP Fabrication

A TPP system was used to fabricate 3D structures using the prepared GelMA hydrogel. The TPP system consisted of a titanium sapphire femtosecond laser (120 fs, 80 MHz, 780 nm Spectra-Physics, Milpitas, CA, USA), beam expansion system, high-precision piezoelectric platform, and charge-coupled device monitoring system. Photosensitive GelMA hydrogel solution (20 μL) was added to a glass substrate using a pipette. A cover glass was used to cover the solution to prevent water evaporation owing to long-term air exposure. A 100 μm thick plastic shim was placed between the glass substrate and cover glass to create the fabrication space. The femtosecond laser was focused on the solution using an oil-immersion focusing objective (60× N.A. 1.42, Nikon, Tokyo, Japan), and 3D microstructures were fabricated via the movement of the laser beam, which was achieved by moving the piezoelectric platform. After the 3D microstructures were fabricated, the unpolymerized hydrogel solution was rinsed with deionized water. The morphological characteristics of the fabricated 3D microstructures were observed using a scanning electron microscopy (SEM; SU-8020, Hitachi, Tokyo, Japan) instrument. The surface roughness of the 3D microstructures was measured using a white light interference 3D surface profiler (NT1100, Wyko, South GreenBack, TN, USA). The polymer line resolution and effect of the hydrogel concentration on the properties of the fabricated 3D scaffolds were analyzed. Subsequently, a 2 mm × 1.73 mm × 0.67 mm GelMA hydrogel three-layer spider web structure was fabricated.

### 2.4. Cell Attachment of 3D Scaffolds

The 3D microstructures fabricated using photosensitive GelMA hydrogel solutions with concentrations of 5%, 8%, 10%, 12%, 15%, and 20% (*w*/*v*) were added to a 75% ethanol (Fuchen, Tianjin, China) solution, ultrasonically cleaned, and sterilized under UV light for 2 h. CCC-ESF-1 human skin fibroblasts were seeded in 6-well plates (3516, Corning Costar, Alexandria, VA, USA) at a density of 2000 cells per well. Subsequently, samples of the fabricated 3D microstructures were placed in the wells that contained cell suspension. The 6-well plates were incubated at 37 °C under a 5% CO_2_ atmosphere for 1, 2, 3, 4, and 5 d. Cell attachment [32] and growth on the 3D microstructures was observed directly using an optical microscope (IX71, Olympus, Tokyo, Japan).

## 3. Results and Discussion

Cells were cultured on the 3D scaffolds fabricated using photosensitive GelMA hydrogel solutions. The biological properties of the materials used to fabricate scaffolds are very important for tissue engineering applications. Hence, in this study, the biological properties of the photosensitive GelMA hydrogel solutions were evaluated by assessing their cytotoxicity, swelling ratio and degradation ratio. Moreover, because hydrogel processing is challenging, the processability of the photosensitive GelMA hydrogel solutions was also investigated. Furthermore, the effects of the hydrogel concentration on the properties of the 3D scaffolds and the biocompatibility of 3D scaffolds fabricated using GelMA hydrogel solutions with different concentrations were investigated.

### 3.1. Biological Properties of GelMA Hydrogel Solutions

**Figure 1:**
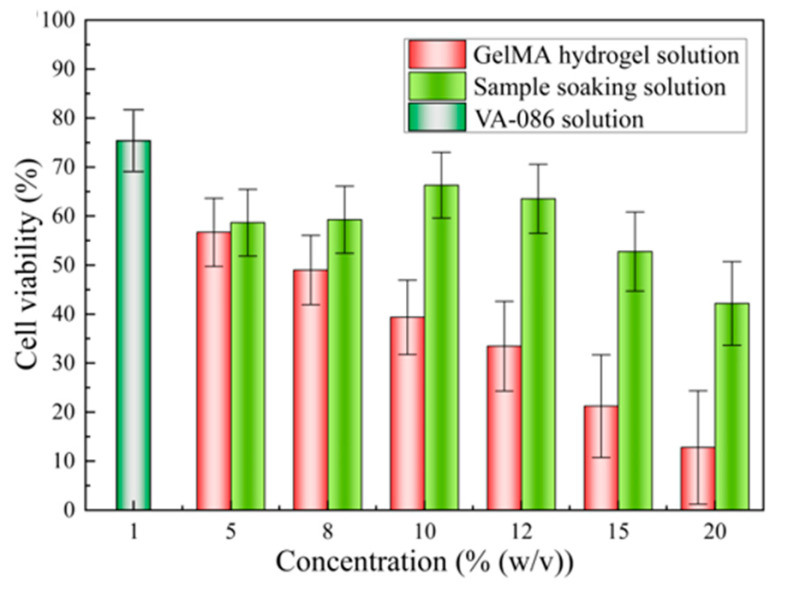
Cytotoxicity of gelatin methacrylate (GelMA) hydrogel, soaking, and VA-086 photoinitiator solutions.

Cytotoxicity: Cytotoxicity is an important parameter for assessing the biological properties of materials. For this experiment, we used a multilabel plate reader with an excitation wavelength of 450 nm. The cytotoxicity of the photosensitive GelMA hydrogel, soaking, and VA-086 photoinitiator solutions were evaluated, and the results are presented in Figure 1. The red bars in Figure 1 indicate the cytotoxicity of the photosensitive GelMA hydrogel solutions with concentrations of 5%, 8%, 10%, 12%, 15%, and 20% (*w*/*v*). Cell activity decreased with increasing GelMA hydrogel concentration and reached a maximum of 56.5% at the GelMA hydrogel concentration of 5% (*w*/*v*). As the GelMA hydrogel concentration further increased, the amount of macromers in solution gradually increased. The cytotoxicity of unpolymerized macromers was higher than that of the polymerized samples, which led to a decrease in cell activity. The lime-green bars in Figure 1 illustrate the cytotoxicity of the solution used to soak the photopolymerized sample. Cell activity first increased and then decreased with increasing GelMA hydrogel concentration of the soaked samples and reached a maximum value of 66.3% when the GelMA hydrogel concentration of the soaked sample was 10% (*w*/*v*). This was attributed to the difference in polymerization degree between the photopolymerized samples. The polymerization degree of the samples fabricated using photosensitive GelMA hydrogel solutions with relatively low concentrations was low. Dense polymer networks were not formed, and a fraction of the long-chain polymers on the edges of the samples were broken. The presence of oligomers in the soaking solution caused an increase in solution cytotoxicity. The polymerization degree increased with increasing GelMA hydrogel concentration, and the concentration of oligomers in the soaking solution gradually decreased. When the GelMA hydrogel concentration was 10% and 12% (*w*/*v*), the effects of the GelMA hydrogel concentration and polymerization degree on cell activity were balanced. However, when the GelMA hydrogel concentration exceeded 12% (*w*/*v*), more organic groups were present in solution. Consequently, soaking solution cytotoxicity increased, which caused a decrease in cell activity. Furthermore, the cytotoxicity of the VA-086 photoinitiator was tested. Because the initiation efficiency of the water-soluble azo photoinitiator was high, photopolymerization was efficiently initiated using a low concentration of photoinitiator. The decomposition products of the azo initiator were non-toxic. Hence, the cell activity of the VA-086 photoinitiator reached 67.1%. A cell activity higher than 50% indicated that the experimental materials presented good biocompatibility. The aforementioned experimental results revealed that: (1) the unpolymerized photosensitive GelMA hydrogel solution presented a certain degree of cytotoxicity, (2) the cytotoxicity of the photosensitive GelMA hydrogel solution significantly decreased after the polymerization of the organic groups, and (3) the photosensitive GelMA hydrogel solutions with concentrations of 10% and 12% (*w*/*v*) were the most suitable for 3D scaffold fabrication because the corresponding polymerized samples presented the lowest cytotoxicity among all analyzed samples.

**Figure 2:**
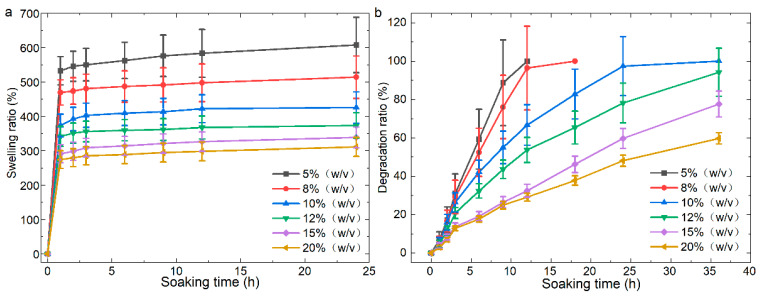
(**a**) Swelling and (**b**) degradation ratios of polymerized gelatin methacrylate (GelMA) hydrogel material samples as the function of soaking time at different GelMA hydrogel solution concentrations.

Swelling ratio: Cell division and growth require water, and the swelling ratio can be used to characterize the water absorption capacity of materials. The swelling ratios of the analyzed samples as a function of soaking time at different GelMA hydrogel concentrations are presented in Figure 2a. After soaking, the swelling ratios of the samples decreased significantly with increasing GelMA hydrogel concentration. The sample fabricated using the GelMA hydrogel solution with a concentration of 5% (*w*/*v*), presented the highest swelling ratio of 607.6%. The swelling ratio of the sample fabricated using the GelMA hydrogel solution with a concentration of 20% (*w*/*v*), was only 311.3%. Moreover, the swelling ratios of the samples fabricated using GelMA hydrogel solutions with the same concentration increased with increasing soaking time. The swelling ratios of the samples increased sharply within the first hour, and then the rate of increase of the swelling ratio decreased. The lower the concentration of the GelMA hydrogel solution was, the larger the macromers gap of the photopolymerized samples was. This is why more water was absorbed when the concentration of the GelMA hydrogel solution was lower. By contrast, the polymer network of the samples fabricated using GelMA hydrogel solutions with higher concentrations was denser and their molecular gap was smaller. Hence, less water was absorbed by these samples, and their swelling ratios were lower.

Degradation ratio: For tissue engineering, cells are cultured on pre-processed 3D scaffolds. The 3D scaffolds should provide support for cells and a microenvironment that is beneficial for cell growth. However, the tissue can be cultivated on artificial 3D scaffolds, which should present a certain degree of degradability. The degradation ratios of the samples fabricated using GelMA hydrogel solutions with different concentrations as a function of soaking time are illustrated in Figure 2b. The degradation ratios of the sample fabricated using GelMA hydrogel solutions with the same concentration increased with increasing soaking time. The degradation ratios of the samples fabricated using GelMA hydrogel solutions with low concentrations were significantly higher than those fabricated using GelMA hydrogel solutions with high concentrations. The sample fabricated using a GelMA hydrogel solution with a concentration of 5% (*w*/*v*) was completely degraded after 12 h of soaking. Protease solutions mainly degrade gelatin. The long-chain structure of low-concentration polymers facilitated full contact between gelatin and the protease solution, which led to a high degradation ratio. When the concentration of the GelMA hydrogel solution used for fabrication the sample was increased to 20% (*w*/*v*), the degradation ratio of the sample after 36 h was only 59.77%. The dense network structure of high-concentration polymers hindered the contact between gelatin and the protease solution inside the polymer network, which caused the degradation ratio to decrease. The swelling and degradation ratio data indicated that: (1) the polymerized GelMA hydrogel material presented excellent water absorption capacity, (2) the gelatin in the polymerized GelMA hydrogel material can be fully degraded, and (3) the photosensitive GelMA hydrogel is suitable for fabricating scaffolds for tissue engineering.

### 3.2. Resolution of Polymer Lines

**Figure 3:**
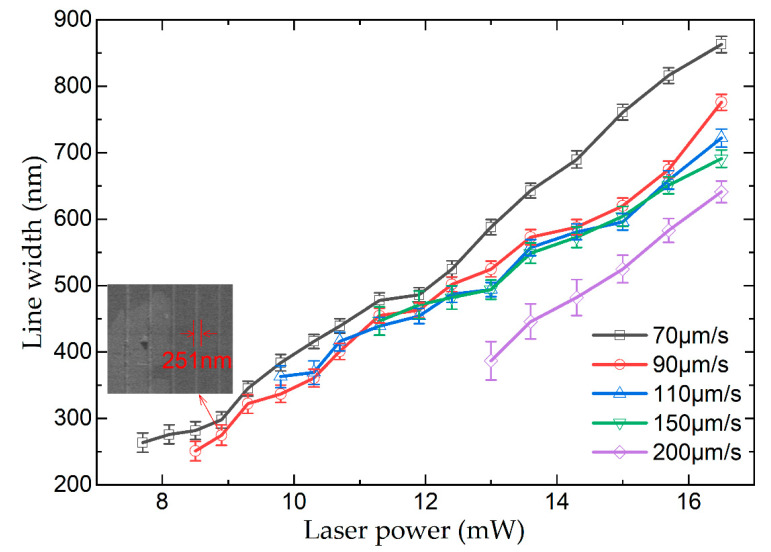
Line width dependence of laser power of polymer lines fabricated using the photosensitive GelMA hydrogel solution with a concentration of 10% (*w*/*v*).

Polymer line scanning is one of the main methods used to assess material processability, and the minimum resolution can be obtained using the line width of the polymer lines. The effects of laser power and scanning speed on the polymer lines fabricated via TPP were analyzed. The photosensitive GelMA hydrogel solution with a concentration of 10% (*w*/*v*) was used to fabricate polymer lines. The laser scanning speed was set at 70, 90, 110, 150, and 200 μm/s. The laser power was gradually reduced at each laser scanning speed, and the minimum processing thresholds were 7.7, 8.5, 9.8, 11.3, and 13 mW at the laser scanning speeds of 70, 90, 110, 150, and 200 μm/s, respectively. The corresponding minimum widths of the polymer lines were 264, 251, 363, 447 nm, and 387 nm, respectively. The dependence of the width of polymer lines fabricated using different laser scanning speeds on the laser power is illustrated in Figure 3. And the SEM image of the polymer lines fabricated at a speed of 90 μm/s is inserted in Figure 3. At the same laser scanning speed, the width of the polymer lines increased with increasing laser power. At the same laser power, the width of the polymer lines decreased with increasing laser scanning speed. At a constant laser scanning speed, the energy obtained in the exposed areas decreased with decreasing laser power. Consequently, the TPP absorption section in the material also decreased. When the laser power was constant a low laser scanning speed caused a high overlap of laser pulses in the laser irradiation areas and the exposed areas presented high energies. Hence, the TPP absorption section decreased with increasing laser scanning speed. Owing to the low polymerization degree, the polymer lines deformed. The experimental results revealed that (1) the selected photoinitiator system can efficiently initiate the TPP effect in photosensitive GelMA hydrogel solutions, (2) when the laser scanning speed was 70 and 90 μm/s, the fabricated polymer lines had smaller width and deformation, and (3) when the laser scanning speed and power were 90 μm/s and 8.5 mW, respectively, the minimum resolution of the fabricated material reached 251 nm.

### 3.3. Effect of GelMA Hydrogel Concentration on the Properties of the 3D Scaffolds

**Figure 4:**
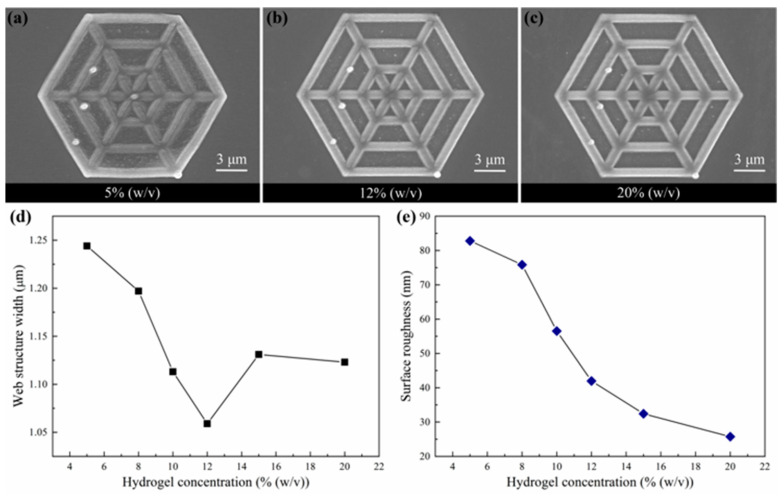
Scanning electron microscopy images of single-layer spider web structures fabricated using photosensitive gelatin methacrylate (GelMA) hydrogel solutions with concentrations of (**a**) 5%, (**b**) 12%, and (**c**) 20% (*w*/*v*) at a laser scanning speed and power of 110 μm/s and 9.5 mW, respectively. (**d**) Width and (**e**) surface roughness of spider web structure dependence on GelMA hydrogel solution concentration.

To analyze the effect of the GelMA hydrogel concentration on the properties of the fabricated 3D scaffolds, the laser power and scanning speed were set to 9.5 mW and 110 μm/s, respectively. The laser power used in this section was lower than that used in Section 3.2 under the same scanning speed. Since the fabrication of the 3D structure required the multiple scanning of laser spots at a fixed overlap rate. The accumulation of energy in the exposure area initiated the TPP effect. Spider web structures were fabricated using GelMA hydrogel solutions with concentrations of 5%, 8%, 10%, 12%, 15%, and 20% (*w*/*v*). The SEM images of the single-layer spider web structures fabricated using the GelMA hydrogel solutions with concentrations of 5%, 12%, and 20% (*w*/*v*) are presented in Figure 4a–c), respectively. The width and surface roughness of the single-layer spider web structures fabricated using GelMA hydrogel solutions with concentrations of 5%, 8%, 10%, 12%, 15%, and 20% (*w*/*v*) were measured, and the results are presented shown in Figure 4d,e, respectively. The width of the spider web structures decreased first and then increased with increasing GelMA hydrogel solution concentration. The spider web structures fabricated using GelMA hydrogel solutions with concentrations of 5% and 12% (*w*/*v*) presented the widest (1.244 μm) and narrowest (1.059 μm) widths, respectively. The surface roughness of 3D structures decreases with increasing hydrogel concentration. The surface roughness values of the spider web structures fabricated using GelMA hydrogel solutions with concentrations of 5% and 20% (*w*/*v*) were the highest (82.81 nm) and lowest (25.73 nm), respectively. The experimental results revealed that: (1) the concentration of photosensitive GelMA hydrogel solution significantly affected the processing accuracy and surface roughness of the fabricated 3D structures, (2) the photosensitive GelMA hydrogel solutions with concentrations of 10% and 12% (*w*/*v*) were the most suitable for fabricating 3D scaffolds because the 3D structures fabricated using these solutions presented high processing accuracy and moderate surface roughness, which facilitated cell growth.

**Figure 5:**
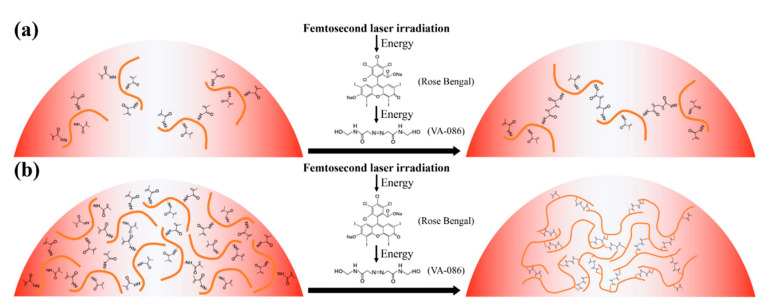
Photopolymerization in photosensitive gelatin methacrylate hydrogel solution at (**a**) low and (**b**) high concentrations.

During the functionalization of gelatin, the ether bonds of methacrylic anhydride broke. Meanwhile, amino groups in gelatin underwent dehydrogenation. Then, GelMA hydrogel macromers were obtained by the synthesis reaction. In polymerization, GelMA macromers combined with each other by methyl groups to form chain polymers. When the concentration of photosensitive GelMA hydrogel solution was low, only a few macromers were present in solution. These macromers could only be polymerized into long-chain polymers (Figure 5a). When the concentration of the photosensitive GelMA hydrogel solution was high, the solution contained sufficient macromers to form dense polymer networks (Figure 5b). Hence, the spider web structure fabricated using a photosensitive GelMA hydrogel solution with a low concentration presented low resistance to deformation. The structure deformed in the longitudinal direction and extended in the transverse direction, resulting in a wide line width. Moreover, long-chain polymers were easily broken during rinsing. The polymers on the surface of the 3D structure were washed away to form irregular stripes. Consequently, the surface of the 3D structure was rough. As the concentration of the photosensitive GelMA hydrogel solution increased, the long-chain polymer structure gradually changed into a network structure. Accordingly, the resistance to deformation of the polymers increased, and the deformation of the spider web structure decreased. Owing to the distribution of the incident laser beam, the polymerization degree of the 3D structure at the edges was low, and the edges of the 3D structure were easily washed away. Hence, the width of the spider web structures decreased as the concentration of the photosensitive GelMA hydrogel solution increased. When the concentration of the photosensitive GelMA hydrogel solution was higher than 12% (*w*/*v*), the density of the polymers further increased with increasing concentration. Furthermore, the spider web structure no longer deformed, and rinsing no longer affected the edges of the 3D structure. Hence, the width of the spider web structure increased and gradually stabilized as the solution saturated. Moreover, the resistance to deformation caused by changes in the polymer structure increased. Hence, the surface roughness of the spider web structure gradually decreased with increasing concentration of the photosensitive GelMA hydrogel solution.

### 3.4. Fabrication of 3D Scaffolds

**Figure 6:**
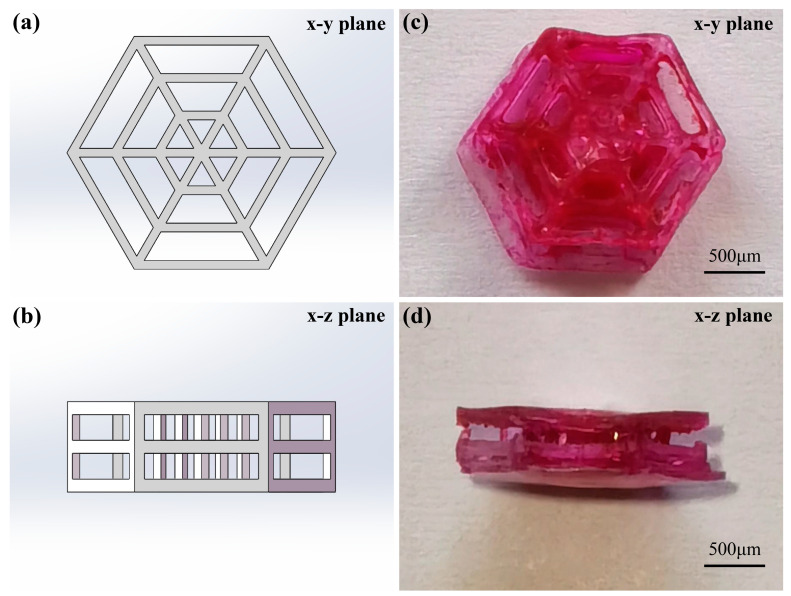
(**a**,**b**) Digital model and (**c**,**d**) photographs of the fabricated 3D scaffold.

Based on the effect of concentration of the photosensitive GelMA hydrogel solution on the properties of the fabricated 3D structures and cytotoxicity test results, a photosensitive GelMA hydrogel solution with a concentration of 12% (*w*/*v*) was used to fabricate 3D scaffolds. A low laser scanning speed ensures a high processing accuracy; however, it causes two drawbacks during fabrication. A long fabrication time causes water to evaporate from the photosensitive GelMA hydrogel solution during fabrication. Moreover, because 3D structure fabrication requires multiple scanning steps, a low laser scanning speed causes the accumulation of an excess amount of energy in the exposure area, which damages the fabricated materials. Consequently, the fabricated 3D structures are incomplete. A high laser scanning speed causes a low processing accuracy and significant deformation of the fabricated 3D structures. Therefore, the laser scanning speed and power used in this study were appropriately adjusted according to the results of the polymer line experiments. The laser scanning speed of 150 μm/s could ensure the proper fabrication time and the fabrication accuracy in large-sized 3D structures fabrication. In the study of polymer lines resolution, the laser scanning speed of 150 μm/s was high. When a low laser power was used for a single scan, the energy obtained in the exposure areas was small, resulting in a low degree of polymerization of the polymer lines. Hence, the polymer lines could not firmly attach to the substrate or severely deformed after rinsing treatment. The complete polymer lines were obtained when the laser power was higher than 11.3 mW. However, in the fabrication of 3D structures, the polymer lines fabricated by low laser power attached to each other. Hence, they would not deform during the rinsing treatment. The high laser power used to fabricate 3D structures under high-speed scanning would damage the photosensitive solution. Therefore, the large-sized 3D structures with high accuracy could be fabricated with laser power lower than 11.3 mW. The 3D scaffold with a three-layer spider web structure fabricated at a laser scanning speed of 150 μm/s and a laser power of 9.8 mW, with a layer distance set to 150 nm is illustrated in Figure 5. Fibroblasts were approximately 10–20 μm in size. The size of 3D scaffolds should range from hundreds of micrometers to millimeters to provide sufficient space for cell growth. The size of the fabricated 3D scaffold illustrated in Figure 6 was 2 mm × 1.73 mm × 0.67 mm.

### 3.5. Cell Shape on 3D Scaffolds

**Figure 7:**
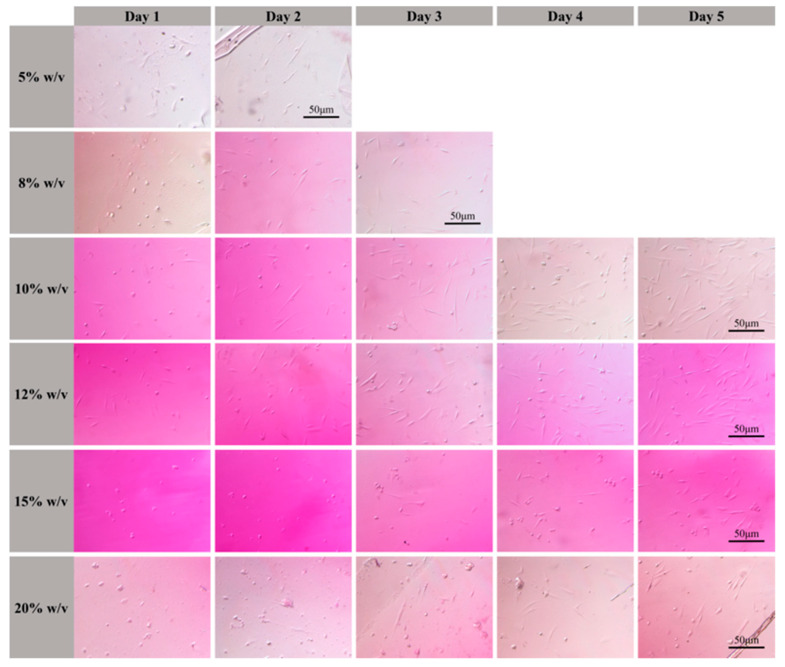
Attachment and growth of CCC-ESF-1 human skin fibroblasts on the 3D scaffolds fabricated using photosensitive GelMA hydrogel solutions with concentrations of 10% and 12% *w*/*v*.

Cell morphology on the fabricated 3D scaffolds was observed and recorded after 1–5 days (Figure 7). Cytotoxicity testing revealed that the solutions after soaking samples fabricated using photosensitive GelMA hydrogel solutions with concentrations of 10% (*w*/*v*) and 12% (*w*/*v*) hydrogel concentration presented a low cytotoxicity, and cells can attach to the 3D scaffolds after 1 d. With prolonged incubation time, cell shape changed from core to spindle, and the number of cells attached to the 3D scaffolds increased. Cell culture on the 3D scaffolds agreed well with the cytotoxicity test results of the photosensitive GelMA hydrogel solutions. The experimental results further confirmed that the photosensitive GelMA hydrogel solutions with concentrations of 10% and 12% (*w*/*v*) were suitable for fabricating tissue engineering scaffolds.

## 4. Conclusions

The fabrication of high-precision scaffolds for tissue engineering with excellent biocompatibility has become a research hotspot. Furthermore, the fabrication of 3D scaffolds using the TPP technology and naturally derived hydrogels provides new prospects in the tissue engineering field. In this study, we systematically analyzed the configuration and biological properties of photosensitive GelMA hydrogel solutions, fabrication of 3D scaffolds, and biocompatibility of the 3D scaffolds. A 780 nm femtosecond laser was used to induce the TPP effect. Photosensitive GelMA hydrogel solutions were configured using combinations of GelMA hydrogel, photoinitiator, and photosensitizer, which ensured the induction of the TPP effect. The biological properties of the materials used to fabricate scaffolds are very important for tissue engineering applications. Hence, in this study, the biological properties of the photosensitive GelMA hydrogel solutions were evaluated using cytotoxicity, swelling ratio, and degradation ratio experiments. Because hydrogel processing is challenging, the processability of the photosensitive GelMA hydrogel solutions was analyzed. The effects of the GelMA hydrogel solution concentration on the properties of the fabricated 3D structures and biocompatibility of 3D scaffolds were also studied. The experimental results indicated that: (1) optimal cell viability was 66.3%, which suggested that the photosensitive GelMA hydrogel solutions presented excellent biological performance, (2) the polymerized GelMA hydrogel presented excellent water absorption and degradation capacities, which suggested that the fabricated 3D structures were suitable for cell attachment, (3) the TPP effect can be efficiently initiated in photosensitive GelMA hydrogel solutions, (4) the concentration of photosensitive GelMA hydrogel solution significantly affected the processing accuracy and surface roughness of the fabricated 3D structures, (5) the photosensitive GelMA hydrogel solutions with concentrations of 10% and 12% (*w*/*v*) were the most suitable for fabricating 3D scaffolds because the high processing accuracy and moderate surface roughness of the fabricated 3D scaffolds facilitated cell growth, and (6) a 3D scaffold with good biocompatibility was fabricated using a laser scanning speed of 150 μm/s, laser power of 7.8 mW, layer distance of 150 nm, and a photosensitive GelMA hydrogel solution with a concentration of 12% (*w*/*v*). High-precision 3D scaffolds with excellent biocompatibility, which can be used in tissue engineering research were fabricated. Due to the low efficiency of femtosecond laser machining, the fabrication of 3D scaffold with nano and micro topographical requirements needs further study by femtosecond laser pulse shaping.

## Data Availability

The data presented in this study are available on request from the corresponding authors.
